# A novel algorithm to predict bone changes in the mouse tibia properties under physiological conditions

**DOI:** 10.1007/s10237-019-01266-7

**Published:** 2019-11-30

**Authors:** Vee San Cheong, Ana Campos Marin, Damien Lacroix, Enrico Dall’Ara

**Affiliations:** 1 Department of Mechanical Engineering, University of Sheffield, Sheffield, UK; 2 Department of Oncology and Metabolism, University of Sheffield, Sheffield, UK; 3 Insigneo Institute for in silico Medicine, University of Sheffield, Sheffield, UK

**Keywords:** Bone remodelling, Finite element analysis, In vivo micro-computed tomography, Bone adaptation, In silico simulation, Validation

## Abstract

Understanding how bone adapts to mechanical stimuli is fundamental for optimising treatments against musculoskeletal diseases in preclinical studies, but the contribution of physiological loading to bone adaptation in mouse tibia has not been quantified so far. In this study, a novel mechanistic model to predict bone adaptation based on physiological loading was developed and its outputs were compared with longitudinal scans of the mouse tibia. Bone remodelling was driven by the mechanical stimuli estimated from micro-FEA models constructed from micro-CT scans of C57BL/6 female mice (*N* = 5) from weeks 14 and 20 of age, to predict bone changes in week 16 or 22. Parametric analysis was conducted to evaluate the sensitivity of the models to subject-specific or averaged parameters, parameters from week 14 or week 20, and to strain energy density (SED) or maximum principal strain (*ε*_maxprinc_). The results at week 20 showed no significant difference in bone densitometric properties between experimental and predicted images across the tibia for both stimuli, and 59% and 47% of the predicted voxels matched with the experimental sites in apposition and resorption, respectively. The model was able to reproduce regions of bone apposition in both periosteal and endosteal surfaces (70% and 40% for SED and *ε*_maxprinc_, respectively), but it under-predicted the experimental sites of resorption by over 85%. This study shows for the first time the potential of a subject-specific mechanoregulation algorithm to predict bone changes in a mouse model under physiological loading. Nevertheless, the weak predictions of resorption suggest that a combined stimulus or biological stimuli should be accounted for in the model.

**Electronic supplementary material:**

The online version of this article (10.1007/s10237-019-01266-7) contains supplementary material, which is available to authorized users.

## Introduction

There is an increasing burden of musculoskeletal diseases such as osteoporosis, osteoarthritis and bone metastases with an ageing society. These diseases disrupt the healthy bone remodelling in bone, increasing the risk of fractures through a reduction in bone mass, quality and/or abnormal loading patterns (Webster et al. 2012). For example, osteoporosis is characterised by the reduction in bone mineral density (BMD), the deterioration of the trabecular architecture, and the thinning of cortical shell, as the resorption of bone tissues by osteoclasts and the addition of new bone by osteoblasts are no longer at equilibrium (Jang and Kim 2010; Müller 2005). Currently, the development of new treatments for diseases relies on preclinical interventions on cell cultures and animal models. A computational model of bone remodelling can be used to test novel interventions in silico and speed up the discovery-to-market time and reduce the cost of novel interventions (Pereira et al. 2015; Schulte et al. 2013b).

Bone remodelling is driven by both biological and mechanical factors in a multi-faceted physiological process to cause bone apposition and resorption (Zadpoor 2013). However, many studies on mouse models have demonstrated that the mechanical environment is a key determinant of bone remodelling in long bones and vertebrae (Birkhold et al. 2017; Pereira et al. 2015; Webster et al. 2012). The form–function relationship where bone adapts its shape and material properties is referred to as Wolff’s law (Wolff 1892), and many bone mechanistic mechanoregulation models in finite element analysis (FEA) have been developed to relate the mechanical stimuli to the bone adaptation (Cheong et al. 2018a; Pereira et al. 2015; Villette and Phillips 2017). Most of these algorithms are based on Frost’s Mechanostat Theory, where bone apposition occurs above a higher, apposition limit and resorption occurs below a lower, resorption limit (Frost 2001). These algorithms have been applied in continuum FEA to predict the internal architecture of bone (Huiskes et al. 1987), extracortical bone formation (Cheong et al. 2018a), ingrowth in tissue engineered implants (Byrne et al. 2007), and response to external mechanical loading (Pereira et al. 2015) with realistic results.

Micro-FEA models use three-dimensional images obtained by high-resolution scanning modalities such as micro-computed tomography (micro-CT), by converting each voxel classified as bone into a finite element (FE). The dimension of the trabeculae is larger than the image resolution; thus, one of the advantages of micro-FEA is that bone microarchitecture can be intrinsically accounted for. In mouse bones, bone remodelling algorithms have been applied to study changes in the cortical and trabecular bone of the caudal vertebra due to extra-physiological loading, obtained from longitudinal in vivo micro-CT imaging (Levchuk et al. 2014; Schulte et al. 2011; Schulte et al. 2013b). Micro-FEA in the mouse tibia has mainly been limited to predicting the strain and stiffness of the mouse tibia, and correlating the results with time-lapsed testing and digital volume correlation (Birkhold et al. 2017; Giorgi and Dall’Ara 2018; Oliviero et al. 2018; Patel et al. 2014). Bone remodelling in whole murine tibia has only been modelled using tetrahedral elements with an inhomogeneous mesh size, and by comparing the predicted shape and density of the loaded leg to the non-loaded contralateral leg (Carriero et al. 2018; Pereira et al. 2015), under the strong assumption that the control leg did not undergo adaptive changes since the start of the experiment. The spatial match of remodelled regions in the caudal vertebra and tibia found by previous studies was accurate in approximately 50% of the surface (Pereira et al. 2015; Schulte et al. 2013b), but the remodelling parameters were determined for a loaded model, which may be different under physiological loading. This information would be useful to elucidate the contributions of novel interventions on bone remodelling.

The aim of this study was to develop the first bone remodelling algorithm for micro-FEA to predict cortical bone changes in the mouse tibia due to physiological loading, and to validate the results with a longitudinal dataset. The main hypothesis was that the parameters for remodelling can be tuned by comparing the geometries of the bones from the experimental and predicted images after bone adaptation, to provide an accurate prediction of bone spatio-temporal changes. Bone densitometric analyses and local accuracy criteria were conducted to assess the accuracy of the bone remodelling algorithm. This was the first time the contribution of physiological loading to bone remodelling was evaluated in the whole mouse tibia.

## Materials and methods

### In vivo micro-CT scanning

Five 14-week-old female C57BL6/J mice underwent in vivo micro-computed tomography (micro-CT) scans (voxel size: 10.4 μm) of their whole right tibiae at weeks 14, 16, 17, 18, 19, 20, 21 and 22 of age. However, only the images from weeks 14, 16, 20 and 22 were used in this study to determine the effect of age on the bone remodelling parameters, and whether the parameters calibrated from the baseline scans can be applied throughout the study. The mice’s weight ranged from 16 g to 22 g during the experimental study. Details of the experimental approach can be found in Lu et al. (2016) and Lu et al. (2017). All the experimental procedures complied with the UK Animals (Scientific Procedures) Act 1986 and were approved by the local Research Ethics Committee of the University of Sheffield. Post-processing of the images involved rigidly registering the bone scans to a reference bone to obtain a similar alignment for all images of every mouse tibia at each time point. The reference bone was aligned such that the long axis of the mouse tibia was aligned to the longitudinal axis, and the anterior–posterior plane bisected the midpoint of the line joining the centres of the articular surfaces of the medial and lateral condyles (Lu et al. 2016). Thereafter, the growth plates and condyles were removed by cropping out the region corresponding to 80% of the tibial length, measured from the end of the proximal growth plate as detailed in Lu et al. (2017). The precision error of the scanning and registration procedure and the protocol to quantify bone changes through densitometric analysis (Sect. 2.6), have demonstrated errors of less than 3.5%, with an intraclass correlation coefficient of over 0.8 in local bone mineral content (BMC). Details of the reproducibility study can be found in Lu et al. (2016) and Lu et al. (2017).

### Micro-FEA models

The greyscale datasets from weeks 14, 16, 20 and 22 were processed to remove the proximal fibula, as the material properties of the tibio-fibular proximal growth plate and joint are not known, but have been reported in a combined experimental and micro-FEA study to transmit a small proportion of force during loading (Yang et al. 2014). Each image was then binarised, by defining a single-level threshold calculated as the midpoint between the peaks of the background and bone in the histogram (image frequency plot) (Oliviero et al. 2017). The threshold value obtained using automatic segmentation was 556.7 ± 28.9 mg/cc. Due to potential errors in segmenting the bone surface associated with partial volume effect (PVE), lower and higher limits of greyscale values around the threshold value were computed by finding the nearest histogram bin where the number of voxels is at least twice that at the threshold value, to detect the start of the greyscale values corresponding to the bone and background voxels. This region, termed transition zone (TZ) in the manuscript, refers to the region of greyscale values which could represent either bone or background. This approach allowed the inclusion of 2–3 layers of pixels from the surface defined by the threshold to be affected by the bone remodelling process (Supplementary Fig. S1). This thickness corresponds to the size of osteoblasts or osteoclastic penetration depth (20–30 microns) (Müller 2005; Puckett et al. 2008). The subject-specific threshold and TZ computed for each mouse from the baseline scan (week 14 of age) were applied throughout the study.

The segmented images from weeks 14 and 20 were used to build three-dimensional micro-FEA models to obtain the strain distribution, which was used with the bone remodelling algorithm to predict the images at weeks 16 and 22, respectively. All bone voxels with grey values above the threshold, without a transition zone, were converted into linear 8-noded hexahedral elements. Homogenous isotropic material properties were assumed, using an elastic modulus of 14.8 GPa and Poisson’s ratio of 0.3 (Oliviero et al. 2018; Webster et al. 2012). The FEA models acquired from micro-CT scans incorporated the microstructure and structural anisotropy of bone, and previous studies showed good correlation between the measured and simulated stiffness, and local displacements for mouse bones (Christen et al. 2014; Macneil and Boyd 2008; Oliviero et al. 2018).

**Fig. 1:**
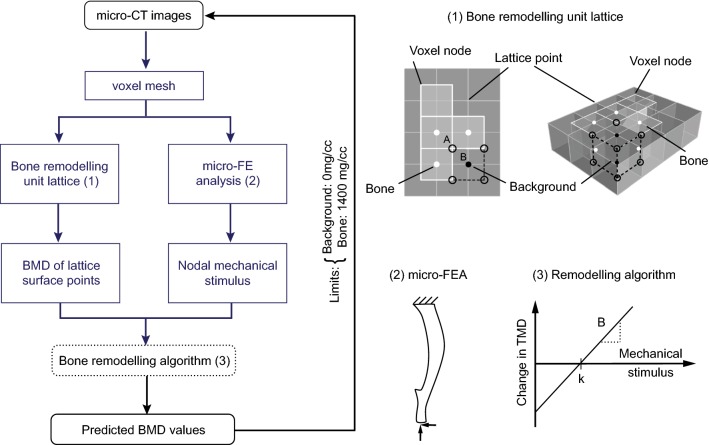
Bone remodelling algorithm flow chart illustrating the generation of virtual micro-CT images at different time points (left). The steps indicated in blue-grey are used in the optimisation algorithm to compute the parameters for bone remodelling. (**1**) Remodelling unit lattice: the stimulus at the voxel node A is used to calculate the new average grey values from the 4 pixels (closed circle, 8 voxels in 3D), which is converted to TMD as it belonged to a bone voxel. A net positive change was applied to the grey value(s) of the background voxel(s) (black closed circle), while negative change was applied to bone voxel(s) (white closed circle). The final value at B was averaged from the contributions of all the 8 open circles. (**2**) Boundary conditions of the micro-FEA model. (**3**) Remodelling algorithm with apposition limit (threshold in mechanical stimulus) *k* and rate of remodelling *B*. Time unit for the change in TMD depends on the time between the acquired micro-CT scans

Three separate FEA analyses using the same model were conducted for each individual mouse, with a 1-N load applied along each anatomical direction (inferior–superior, anterior–posterior, medial–lateral) independently, before scaling the results to the corresponding load value in each direction. This approach allows for simple post-processing of the results in all possible loading scenarios by using the superimposition of the effects, a principle that can be used as the models are linear. All the nodes on the proximal surface of the bone were fully constrained (Fig. 1). The nodes on the distal surface were restrained from any rotation, via kinematic coupling to the area centroid of the distal surface. Peak physiological walking load at the ankle joint was calculated by solving a free-body diagram, using the mass of the foot, and force plate data available in Charles et al. (2018), which recorded an average peak vertical and horizontal ground reaction force of 120% and 10.9% of body mass, respectively. No muscle load was included. The stimulus values were scaled to the body mass (BW) of the mice used in this study (Table 1). Only the stimuli along the superior–inferior and the posterior–anterior directions were included in the superimposition of the results as the medial–lateral force was reported to be much smaller. Sensitivity analysis conducted on the 5 specimens also showed that the inclusion of this additional component did not significantly affect the results (Supplementary Table S1). The FEA models were solved using Abaqus 2017 (Dassault Systèmes Simulia, RI, USA) on the University of Sheffield High Performance Computing Clusters (ShARC).

**Table 1:** Physiological loading applied in the FEA model, per body mass (BW) in grams

	Load/BW (N/g)
Inferior–superior	0.01355
Anterior–posterior	0.00289
Medial–lateral	0

### Algorithm

A quasi-static analysis was conducted assuming that bone remodelling is the outcome of a response to the peak applied stimulus, which has been shown previously to account for the main changes in bone remodelling under daily load history (Huiskes 2000).

As bone remodelling units (BRUs) react to mechanical and biological stimuli at the surface to cause bone remodelling, only the surface nodes were selected for the bone adaption algorithm to be applied. In this approach, no biological stimulus was included and bone remodelling was based on mechanical stimulus alone. A node-based approach for bone remodelling, instead of an element-based method using integration points, was implemented as it minimises the occurrence of checkerboard discontinuities (Chen et al. 2007). The proposed mechanoregulation algorithm in Fig. 1 assumes a linear response to the stimulus (Huiskes et al. 1987), and the same rate of remodelling in both apposition and resorption. No lazy zone without remodelling (i.e. a range of stimulus values where no response is induced) was modelled initially, following the results obtained for the mouse caudal vertebra and the human distal tibia (Christen et al. 2014; Razi et al. 2015; Schulte et al. 2013a). The effect of including the lazy zone was later investigated as part of the parametric analysis. The nodal values from the FEA outputs were used to compute the mean change in the grey values of the voxel density, according to the following equation, which was converted to tissue mineral density (TMD) according to the linear mathematical relationship provided by the CT manufacturer for bone voxels:

1 $$\Delta {\text{TMD}}\left( N \right) = B\left( { {{\varTheta }}\left( N \right) - k} \right)$$

where *B* is the rate of bone remodelling, *k* is the chosen threshold of the mechanical stimulus, and *Θ* is the actual mechanical stimulus in the node *N*. The nodal values were obtained by extrapolating the values from the integration points to the nodes before averaging them. Strain energy density (SED) and maximum principal strain (*ε*_maxprinc_) were used as the stimulus separately as they have been shown to give realistic results in bone remodelling simulations (Cheong et al. 2018b; Schulte et al. 2011; Villette and Phillips 2017).

Each BRU was defined to be of the same size as the image voxel and on the same plane as the image slice, with the midpoint of its lattice face coincident with the node of the voxel. Hence, the vertices of each BRU (lattice point) were coincident with the centres of the top/bottom face of 8 voxels (or 4 pixels in 2D) (Fig. 1). The new average tissue mineral density (TMD) at the surface node for the following iteration was computed by summing the change in density at the surface node and the averaged TMD of the surrounding 8 voxels. The change in density was applied only to either ‘bone’ or ‘surface’, by linearly scaling their grey values by a fixed value, so that the average of all voxels was equivalent to the predicted TMD at the next iteration. For example, resorption was predicted when there was an overall negative change, and the scaling factor was applied only to all voxels labelled as ‘bone’. The TZ region was allowed to undergo both resorption and apposition, under the assumption that there are both osteoclasts and osteoblasts acting on the surface. Depending on the magnitude of the mechanical stimulus in that region, apposition or resorption was defined. The TMD values from all the connected BRUs at each voxel were averaged, to compute a final BMD value, which was used to update the images for the following iteration. As the mechanoregulation algorithm used has an open-loop control, the limits of TMD values for the predicted images were set at 0 and 1400 mg/cc before the images were converted back to grey values, to ensure that the final TMD values remained realistic (Fig. 1). The upper threshold was set by first identifying the peak TMD value of bone voxels in the histogram, and choosing a value slightly higher than that.

### Parameter selection

The proposed mechanoregulation algorithm assumes that bone adapts to physiological loading according to the apposition limit (threshold in the mechanical stimulus), *k,* and rate for bone remodelling, *B*. This changes the grey values of the bone and background voxels, which affects which voxels are segmented as bone in the next iteration. This resulting change in bone geometry affects the structural properties observed in the next time point, in particular the bending stiffness. The results from a previous study using the same dataset showed that the FEA predicted experimental stiffness increases with age, and that the normalised FEA predicted stiffness at week 22 was 14.1 ± 1.9% higher than that at week 14 (Lu et al. 2017). In this study, a time step of 2 weeks was used. From classical mechanics, the bending stiffness is a function of the object’s second moment of area or volumetric second moment (Hibbeler 2005). As the length of mouse tibia increases slightly with age, the volumetric second moment, *I,* was used to account for differences in tibia length, by dividing the tibia into 10 sections according to Lu et al. (2016):

2 $$I_{{{\text{xx}}\left( {\text{vol}} \right)}} = {\iint }y^{2} {\text{d}}V$$

3 $$I_{{{\text{yy}}\left( {\text{vol}} \right)}} = {\iint }x^{2} dV$$

**Fig. 2:**
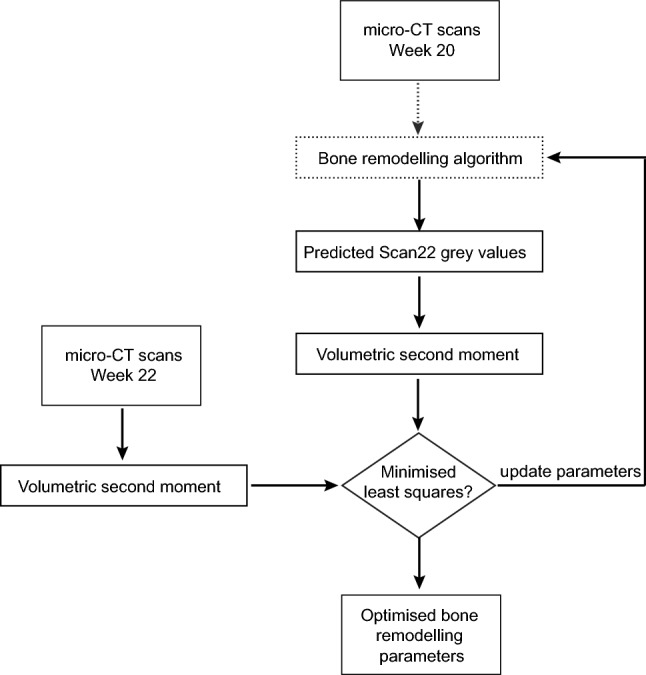
Optimisation algorithm to calculate the subject-specific parameters for bone remodelling, illustrated for weeks 20–22 here. The same algorithm is applied similarly to obtain the parameters between weeks 14–16. The steps indicated by dotted arrow are represented in detail by the blue-grey steps in Fig. 1

Hence, by minimising the least squares of the volumetric second moment between the experimental scan at the next time point (weeks 16 and 22) and the predicted bone, *B* and *k* could be estimated for each subject (Fig. 2):

$$\min\,r \left( B_{j} ,k_{j} \right) = \mathop \sum \limits_{i = 1}^{n} \left( I_{\text{xx} , j + 1} - I_{\text{xx(predicted)} , j }\left( B_{j} ,k_{j} \right) \right) + \mathop \sum \limits_{i = 1}^{n} \left( I_{\text{yy},j + 1} - I_{\text{yy(predicted)}, j} \left( B_{j} ,k_{j} \right) \right)$$

4 $${\text{subject}}\, {\text{to}}\, B_{j} > 0\, {\text{and}}\, k_{j} > 0$$

where *i* is the bone section under consideration, *n* is the total number of sections in the bone, and *j* is the time point being analysed.

The values of *B* and *k* were calculated using sequential quadratic programming, a constrained nonlinear optimisation technique (MATLAB 2018A, The MathWorks Inc., Natick MA, USA), to prevent non-negative values for the volumetric second moment (Christen et al. 2012). A grid search using 100 initial parameters was conducted to ensure that the solutions found represented the global optima.

### Parametric analysis

Four sensitivity analyses were conducted to predict the images at week 22 of age as follows:

1. Subject-specific parameters calibrated between weeks 20–22 without lazy zone.
2. Averaged parameters calibrated between weeks 20–22 without lazy zone.
3. Subject-specific parameters calibrated between weeks 20–22 with a lazy zone.
4. Subject-specific parameters calibrated between weeks 14–16 without lazy zone.

The lazy zone was assumed to be symmetrical about the apposition limit, and it was computed by including it as an extra parameter to be calculated in the optimisation procedure.

### Output measurements and comparison of experimental and numerical predictions

Densitometric analyses were conducted on the experimental and predicted scans by calculating the bone volume (BV), bone volume fraction (BV/TV), bone mineral content (BMC) and volumetric bone mineral density (BMD). BV sums the total volume of bone voxels obtained after binarisation into bone and background, whereas TV is the total volume enclosed by the periosteal surface. The local tissue mineral density (TMD) is evaluated in each voxel with the densitometric calibration of the grey values in the micro-CT images. The total BMC or the BMC in a region is computed as the sum of the TMD in the region, followed by a multiplication of the total voxel volume. BMD is computed by normalising BMC by the total volume (TV) identified by the periosteal surface. The analyses were conducted separately in 40 regions where the bone was divided into 4 compartments (anterior, lateral, medial, posterior) and 10 longitudinal sections (Lu et al. 2016). In addition, the difference between the predicted and experimental bone densitometric indices at week 22 was computed to estimate the error (Schulte et al. 2011). To ensure that the same length of bone is compared, a two-step procedure was utilised. Firstly, the experimental week 22 images were rigidly registered to the week 20 images by centring their volume centroids. The main axis remained unchanged as both images were already rigidly registered to a reference bone. Thereafter, a bounding box was applied to obtain the same number of slices and dimensions for both scans.

To compare the predictive abilities of the model, the amount of overlap between the binarised predicted and experimental scans was computed, after registration and binarisation of the images as described above. The overlap ratio was defined as the intersection of the binarised predicted and the experimental scan, normalised by the total area occupied by both scans (Eq. 5):

5 $$\frac{ {\text{Predicted week 22}} \cap {\text{Experimental week 22}} }{{\text{Predicted week 22}} \cup {\text{Experimental week 22}}}$$

Thereafter, the simulated and experimental spatial patterns of bone apposition and resorption were computed, to ascertain the amount of surface remodelling. The amount of apposition and resorption was computed by comparing the differences between the grey values of the two images, using Eq. 6, for both endosteal and periosteal surfaces:

6 $$\left( \text{Predicted week 22}{-}\text{Experimental week 20} \right) \cap \left( \text{Experimental week 22}{-}\text{Experimental week 20} \right)|\text{voxels} \in \text{bone}\,\text{surface}$$

To compare the accuracy and precision of the prediction, two metrics were used. Firstly, the spatial match, defined as the predicted voxels matching the experimental sites, was computed by normalising Eq. 6 by the predicted amount of bone remodelling. Secondly, experimental sites predicted by the model were calculated as the ratio between Eq. 6 and the actual amount of bone remodelling (measured experimentally). The Wilcoxon signed-rank test was used to test for any significant difference (p < 0.05) (Origin 2018, OriginLab Corp., Northampton, MA).

## Results

### Effect of bone remodelling and parameters of the remodelling algorithm

The regions of high strain energy density (SED) were similar at week 14 and week 20 under 1N load (Supplementary Fig. S2). For superior–inferior load, the regions of high SED were located at the interosseous crest and at the distal anterior section. For 1N anterior–posterior load, high SED values above 0.01 MPa were found near the boundary conditions. In the proximal top half of the tibia, high SED values were also found near the proximal tibial crest, tibial ridge and the interosseous crest. Distally, they were found in both the anterior and posterior regions. For both loading conditions, the regions of highest SED remained unchanged, but the areas decreased from week 14 to week 20.

The displacements on the distal surface under anterior–posterior loading for the week 14 mice were 0.00888 ± 0.00053 mm, 0.00046 ± 0.00018 mm and 0.00132 ± 0.00016 mm in the anterior–posterior, medial–lateral and superior–inferior directions, respectively. Under axial load, the displacements on the distal surface were 0.00621 ± 0.00073 mm, 0.00489 ± 0.00130 mm and 0.00205 ± 0.00017 mm in the anterior–posterior, medial–lateral and superior–inferior directions, respectively.

The percentage change in the second moment of volume followed a concave shape except for *I*_xx_ at week 14. The highest increase was in the middle of the bone at both time periods (Sect. 5). Both *I*_xx_ and *I*_yy_ were generally higher between weeks 14–16 than 20–22 in other regions (Supplementary Fig. S3). The rate and threshold of remodelling (apposition limit), calculated by optimising the volumetric second moment for weeks 14–16 and weeks 20–22, are presented in Table 2, and the parameters obtained for each stimulus showed similar ranges for the two time periods used. The apposition limits for SED between weeks 14–16 and 20–22 in Table 2 corresponded to 25.6 and 39.8 μstrain, respectively. The accompanying remodelling rates were approximately 3.60 and 2.21 mg/cc-μstrain between weeks 14–16 and 20–22, respectively, after converting SED to strain. When the lazy zone was included as an additional degree of freedom in the optimisation procedure, the bandwidths around the apposition limits when SED was used as the stimulus were 0.381 ± 0.830 and 0.404 ± 0.665 between weeks 14–16 and 20–22, respectively. The bandwidths obtained using *ε*_maxprinc_ as the stimulus were 0.192 ± 0.157 and 0.167 ± 0.213 between weeks 14–16 and 20–22, respectively.

**Table 2:** Parameters obtained from optimisation that were used in this study

Stimulus	SED		*ε*_maxprinc_	
Parameter	Remodelling rate, *B* (mg/cc-Pa-2 weeks)	Apposition limit, *k* (Pa)	Remodelling rate, *B* (mg/cc-μstrain-2 weeks)	Apposition limit, *k* (μstrain)
Weeks 14–16	0.31 ± 0.18	4.86 ± 5.83	3.34 ± 1.13	27.9 ± 13.1
Weeks 20–22	0.19 ± 0.09	11.7 ± 6.4	2.16 ± 1.80	20.4 ± 15.8

Numbers indicate averaged values every 2 weeks ± standard deviation calculated for the 5 difference mice

### Bone densitometric analysis

**Fig. 3:**
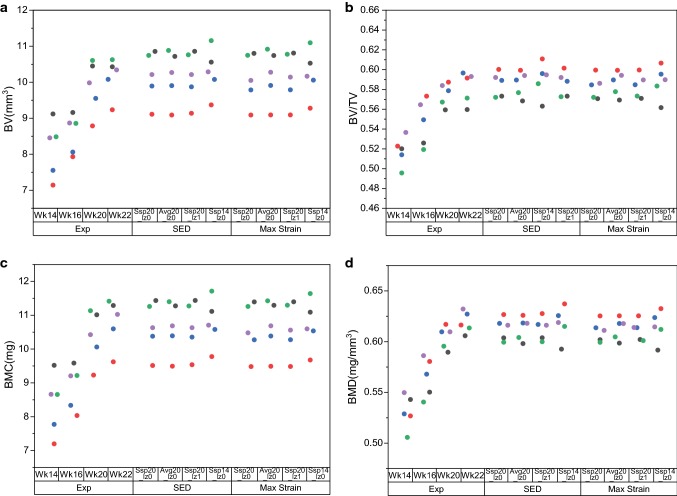
Densitometric indices of experimental (exp) scans of mice at weeks 14, 16, 20 and 22 compared with the simulated results at week 22. SSp20_lz0: subject-specific parameters from weeks 20–22, no lazy zone. Avg20_lz0: averaged parameters from weeks 20–22, no lazy zone. Ssp20_lz1: subject-specific parameters with lazy zone from weeks 20–22, no averaging. Ssp14_lz0: subject-specific parameters from weeks 14–16, no lazy zone. Coloured dots identify the mice in the experiments

The bone density results, calculated for the whole bone, showed no significant difference for the experimental scans between weeks 20 and 22 (Fig. 3, p > 0.05). The predicted results were also very similar for all tested parameters and stimuli. Hence, all subsequent analyses were conducted separately on 40 compartments in the bone.

**Fig. 4:**
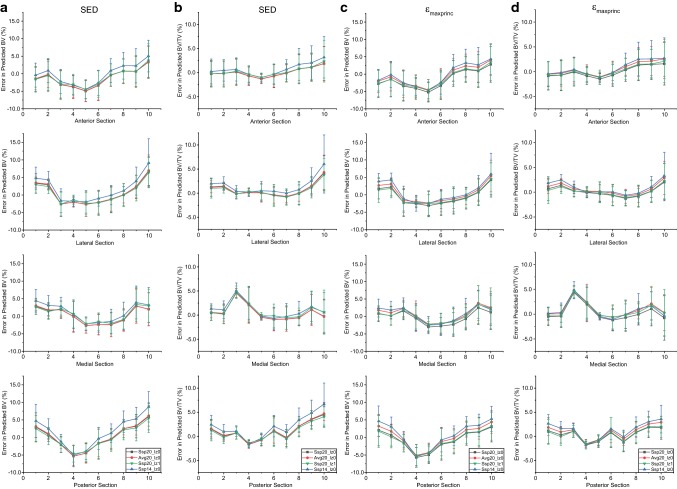
Errors in predicted BV and BV/TV for (**a**–**b**) SED and (**c**–**d**) *ε*_maxprinc_ across the 40 sections of bone. Ssp20_lz0: subject-specific parameters from weeks 20–22, no lazy zone. Avg20_lz0: averaged parameters from weeks 20–22, no lazy zone. Ssp20_lz1: subject-specific parameters from weeks 20–22, with lazy zone. Ssp14_lz0: subject-specific parameters from weeks 14–16, no lazy zone. X-axis indicates longitudinal sections from distal (0) to proximal (10)

The densitometric values increased from weeks 14 to 22, and the changes between weeks 14–16 and 20–22 were small (Supplementary Fig. S4). The errors in predicted BV across the 10 sections followed a concave shape for both mechanical stimuli tested (Fig. 4). There was an over-prediction in BV for the proximal and distal regions, and an under-prediction in the diaphysis. The errors in predicted BV/TV followed a similar pattern, with a spike in section 3 of the medial compartment. However, in the SED case, this error was approximately half of the absolute highest error, which was located in section 10 of the lateral compartment (Table 3). The BMC and BMD results followed the same trends as the BV and BV/TV results and are only included in the Supplementary Material Fig. S5. The curves obtained using calibrated images from weeks 20–22 were insensitive to the parameters tested and resulted in errors of approximately 10%, but the use of parameters calibrated from images between weeks 14–16 introduced errors of up to 15% for both choices of stimulus. The absolute highest error was located in section 10 of the lateral compartment. While the other errors were mainly confined to section 5 of the anterior section for SED, the absolute highest errors were also located in sections 4 and 8 of the medial compartment for *ε*_maxprinc_. The results were not statistically significantly different for all cases (*p* > 0.05).

**Table 3:** The location within the 40 partitions of the tibia (10 longitudinal sections, 4 sectors for each section) with the absolute highest error

Stimulus	Error type	Parameter	Compartment	Section	Absolute highest error (%)
SED	BV	Ssp20_lz0	Posterior	10	12.3
		Avg20_lz0	Lateral	10	13.9
		Ssp20_lz1	Posterior	10	12.3
		Ssp14_lz0	Lateral	10	19.1
SED	BV/TV	Ssp20_lz0	Lateral	10	10.3
		Avg20_lz0	Lateral	10	9.9
		Ssp20_lz1	Lateral	10	9.3
		Ssp14_lz0	Lateral	10	14.5
SED	BMC	Ssp20_lz0	Anterior	5	10.3
		Avg20_lz0	Anterior	5	10.2
		Ssp20_lz1	Anterior	5	9.7
		Ssp14_lz0	Lateral	10	16
SED	BMD	Ssp20_lz0	Lateral	10	9.1
		Avg20_lz0	Lateral	10	8.7
		Ssp20_lz1	Anterior	10	10.1
		Ssp14_lz0	Lateral	10	13.4
*ε*_maxprinc_	BV	Ssp20_lz0	Lateral	10	10.3
		Avg20_lz0	Lateral	10	12.6
		Ssp20_lz1	Lateral	10	10.4
		Ssp14_lz0	Lateral	10	15.2
*ε*_maxprinc_	BV/TV	Ssp20_lz0	Medial	4	8.1
		Avg20_lz0	Medial	4	8.8
		Ssp20_lz1	Medial	4	8.2
		Ssp14_lz0	Lateral	10	9.9
*ε*_maxprinc_	BMC	Ssp20_lz0	Anterior	5	10.9
		Avg20_lz0	Medial	8	10
		Ssp20_lz1	Anterior	5	10.8
		Ssp14_lz0	Posterior	1	13.1
*ε*_maxprinc_	BMD	Ssp20_lz0	Anterior	10	8.9
		Avg20_lz0	Anterior	10	8.5
		Ssp20_lz1	Anterior	10	8.9
		Ssp14_lz0	Medial	10	10.4

Ssp20_lz0: subject-specific parameters from weeks 20–22, no lazy zone. Avg20_lz0: averaged parameters from weeks 20–22, no lazy zone. Ssp20_lz1: subject-specific parameters from weeks 20–22, with lazy zone. Ssp14_lz0: subject-specific parameters from weeks 14–16, no lazy zone

### Spatial match and accuracy of surface remodelling

**Fig. 5:**
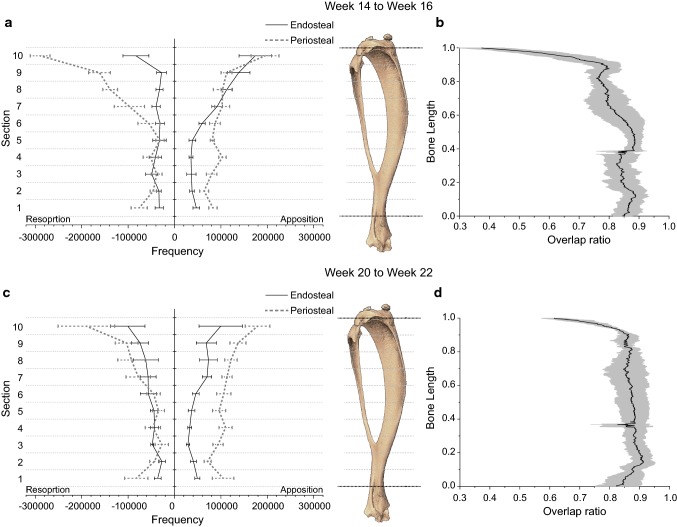
Experimentally measured resorption and apposition of 5 tibiae on the endosteal and periosteal surfaces from (**a**) weeks 14–16 and (**c**) weeks 20–22. Lines indicate average and standard deviation. Overlap ratio across all slices for mouse 5 (**b**) at week 16, using subject-specific parameters from weeks 14–16 images without a lazy zone and (**d**) at week 22, using subject-specific parameters from weeks 20–22 images without a lazy zone. The overlap ratio measured the degree of overlap between the binarised predicted and the experimental scan, normalised by the total area occupied by both scans

The remodelling on the bone surfaces from weeks 20–22, obtained by comparing the experimental images obtained at the two time points, is visualised in Fig. 5. The total remodelling was higher on the periosteal surfaces than the endosteal surfaces. Apposition was higher on the periosteal surfaces for all sections, but resorption was higher on the endosteal surfaces in the diaphysis. For comparison, the apposition were 28.3 ± 16.9% lower and 11.3 ± 10.5% higher than the changes observed between weeks 14–16 on the endosteal and periosteal surfaces, respectively. Resorption were 44.0 ± 53.4% higher and 23.9 ± 11.4% lower than the changes observed between weeks 14–16 on the endosteal and periosteal surfaces, respectively. Overall, the apposition on all bone surfaces was 22.6 ± 11.8% lower than between weeks 14–16, while the resorption was similar between the two time periods (1.3 ± 17.3%).

For all parameters tested for SED and *ε*_maxprinc_, The volumetric overlap between the experimental and predicted week 22 images was 86 ± 3% for the whole bone. Sub-analysis conducted using subject-specific parameters calibrated between weeks 20–22 showed that the overlap was above 75% across the tibial slices, except at two regions – at the proximal end (below the growth plate) and at approximately 40% of the bone length, where a cut of the fibula was made during image processing (Fig. 5). A similar pattern was observed for the overlap between the experimental and predicted week 16 images using parameters calibrated between weeks 14–16, but the value was lower at 80 ± 4%.

The predicted voxels matching with experimental sites (spatial match) for the whole tibia were similar in apposition and resorption (Table 4) for all parameters and stimuli evaluated. The percentage of experimental sites predicted by the model (prediction accuracy) in apposition was higher for SED than *ε*_maxprinc_. The prediction accuracy in resorption was low for both stimuli. *ε*_maxprinc_ was more sensitive to the parameters used than SED.

**Table 4:** Overall accuracy in apposition and resorption for predicted week 22 images

Stimulus		SED		*ε*_maxprinc_	
Parameter	Remodelling measure	Spatial match/%	Prediction accuracy/%	Spatial match/%	Prediction accuracy/%
Ssp20_lz0	Apposition	59.1 ± 3.3	83.6 ± 7.0	59.3 ± 3.2	70.1 ± 11.9
Avg20_lz0	Apposition	59.1 ± 3.3	83.6 ± 6.6	59.2 ± 3.3	81.0 ± 6.5
Ssp20_lz1	Apposition	59.1 ± 3.3	84.0 ± 6.7	59.3 ± 3.2	72.4 ± 12.4
Ssp14_lz0	Apposition	59.1 ± 3.3	84.6 ± 6.4	59.2 ± 3.2	68.7 ± 14.9
Ssp20_lz0	Resorption	47.4 ± 7.0	2.2 ± 0.7	47.2 ± 11.0	13.5 ± 8.9
Avg20_lz0	Resorption	47.2 ± 7.0	2.2 ± 0.3	45.2 ± 5.5	4.5 ± 0.5
Ssp20_lz1	Resorption	48.0 ± 7.6	1.8 ± 0.7	47.2 ± 10.9	12.0 ± 8.8
Ssp14_lz0	Resorption	49.1 ± 7.8	1.4 ± 0.7	45.7 ± 7.3	15.3 ± 14.2

Ssp20_lz0: subject-specific parameters from weeks 20–22, no lazy zone. Avg20_lz0: averaged parameters from weeks 20–22, no lazy zone. Ssp20_lz1: subject-specific parameters from weeks 20–22, with lazy zone. Ssp14_lz0: subject-specific parameters from weeks 14–16, no lazy zone

**Fig. 6:**
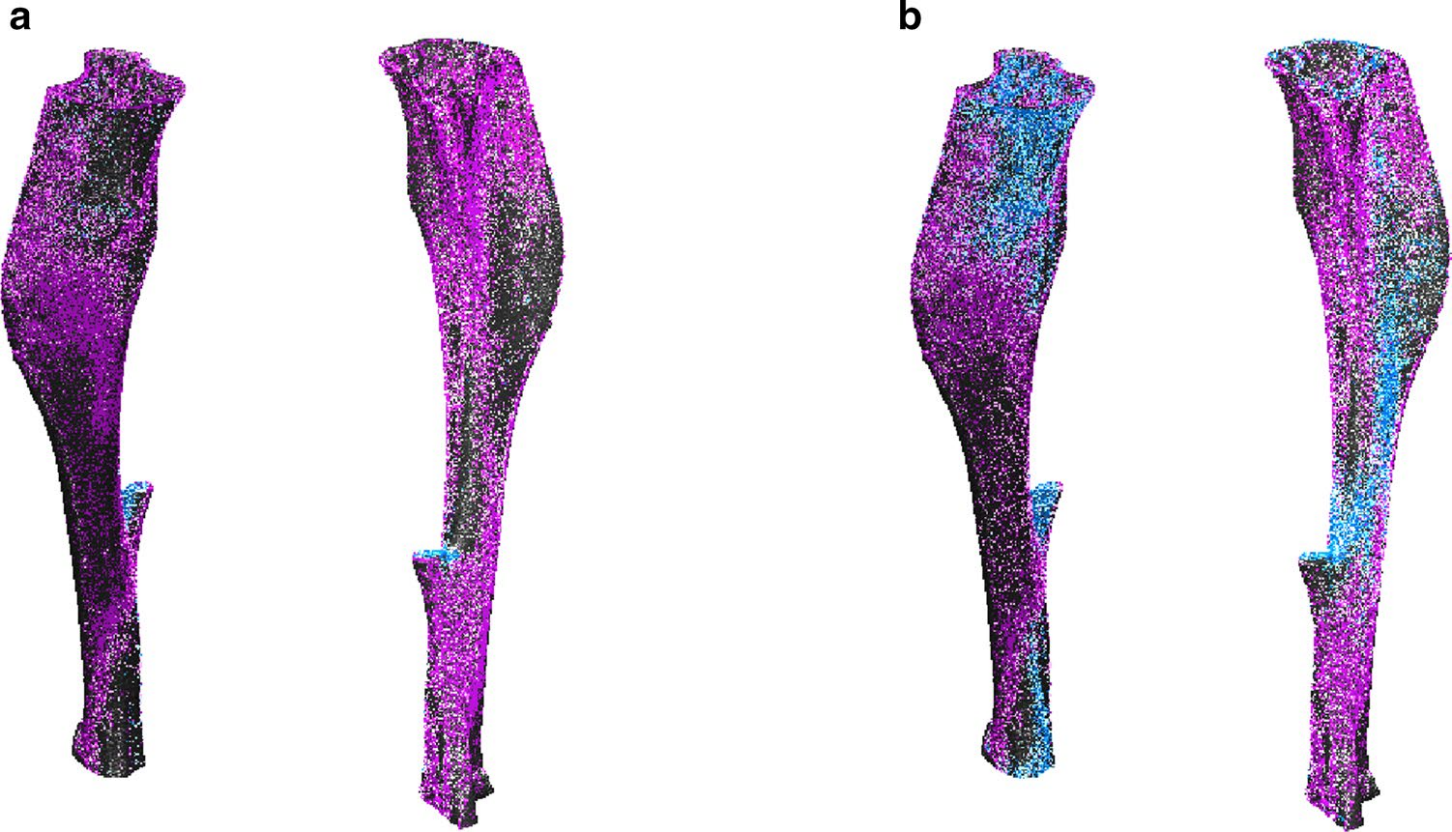
Voxels predicted correctly in apposition (pink) and resorption (blue) for mouse 5 using subject-specific parameters from weeks 20–22 without a lazy zone, overlaid on the experimental scans at week 22 (grey), using (**a**) SED or (**b**) *ε*_maxprinc_ as the stimulus

Figure 6 shows the regions of apposition (pink) and resorption (blue) of mouse 5 predicted correctly by the model using subject-specific parameters from weeks 20–22, overlaid on the 3D reconstruction of the experimental bone from week 22. Using SED as the stimulus captured resorption mainly at the remnants of the fibula, while *ε*_maxprinc_ captured resorption on the anterior-medial proximal tibia, the lateral-posterior diaphysis and the medial distal tibia. The spatial match for *ε*_maxprinc_ was grainy, as the regions of spatial match were more scattered throughout the bone.

**Fig. 7:**
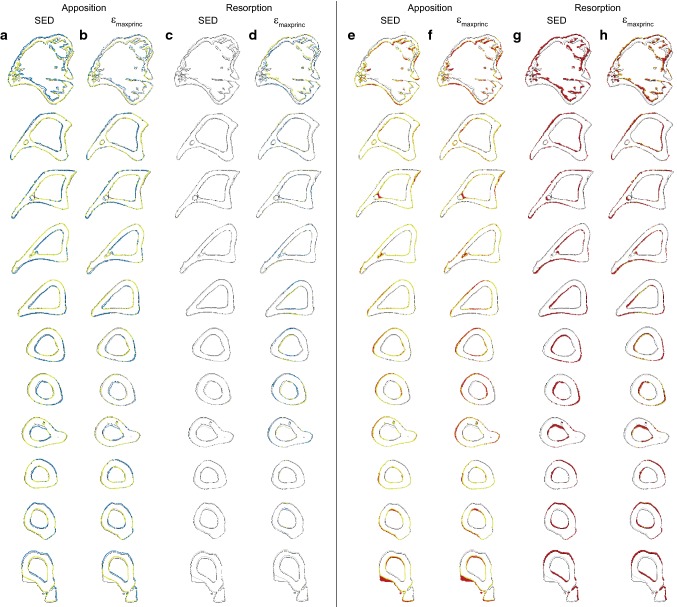
Spatial match (**a**–**d**) and prediction accuracy (**e**–**h**) across 10 sections of mouse 5 using subject-specific parameters from week 20 for (**a**, **e**) apposition in SED, (**b**, **f**) apposition in *ε*_maxprinc_, (**c**, **g**) resorption in SED and (**d**, **h**) resorption in *ε*_maxprinc_. Yellow-green: voxels predicted correctly by the model. Blue: Predicted bone remodelling that did not match with experimental results. Red: Remodelling observed experimentally between weeks 20 and 22 that were not predicted by the model. Grey: Bone contours at week 20. Black: Bone contours at week 22

The spatial match and accuracy of the predicted and experimental results across the 10 longitudinal sections for mouse 5 at week 22, using parameters calibrated from weeks 20–22 without a lazy zone, are shown in Fig. 7. Higher spatial match on the periosteal surface than the endosteal surface for apposition (in yellow-green) was observed, for both SED and *ε*_maxprinc_. The use of SED predicted less resorption than when *ε*_maxprinc_ was used for mouse 5 (in blue).

**Fig. 8:**
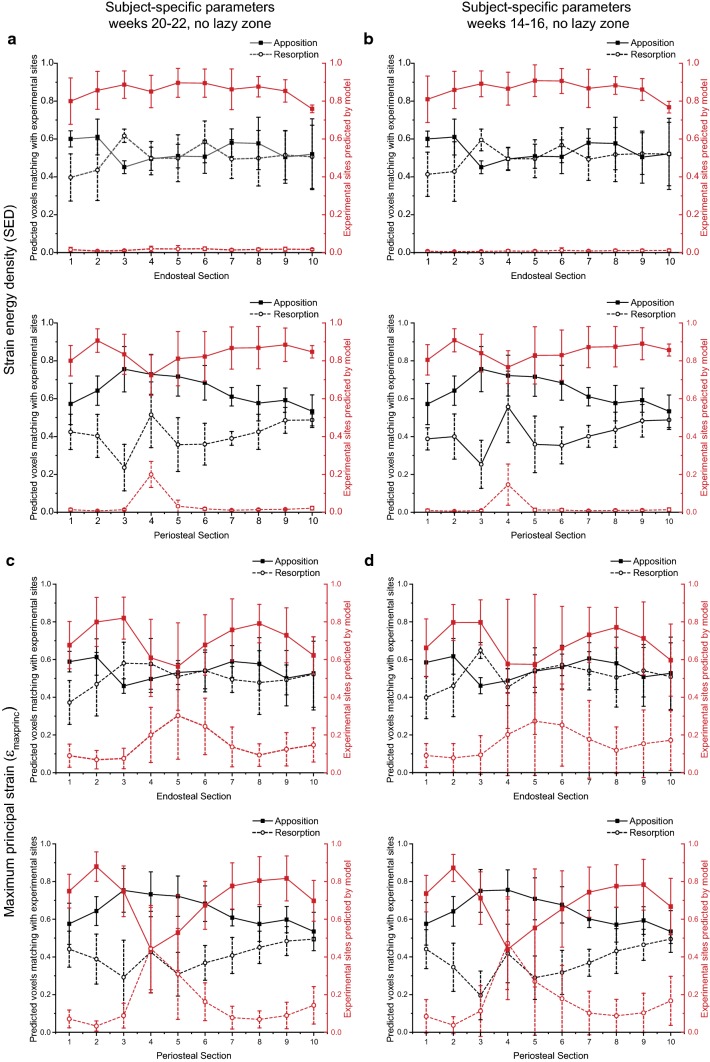
Comparison of the spatial match (left axis) and predictive accuracy (right axis) at the endosteal and periosteal surfaces at week 22, using parameters calibrated without a lazy zone: (**a**) subject-specific parameters from weeks 20–22 (SED), (**b**) subject-specific parameters from weeks 14–16 (SED), (**c**) subject-specific parameters from weeks 20–22 (*ε*_maxprinc_) and (**d**) subject-specific parameters from weeks 14–16 (*ε*_maxprinc_). Solid lines indicate apposition, while dashed lines indicate resorption

Figure 8 displays the spatial match and predictive accuracy at week 22, using subject-specific parameters without a lazy zone calibrated from weeks 20–22 and weeks 14–16 for the endosteal and periosteal surfaces. Similar to the results for the whole bone, the sectional results were not significantly different for the predictions using the parameters calibrated from the weeks 20–22 or weeks 14–16 images for both stimuli (p > 0.05). The predictive accuracy was above 70% for apposition, on both the periosteal and endosteal surfaces. However, there was poor prediction accuracy in resorption, in particular for models based on SED. The prediction accuracy in resorption was higher for *ε*_maxprinc_, but still poor. This was at the expense of a lower predictive accuracy in apposition.

The spatial match in apposition was more evenly predicted on the endosteal surfaces for models based on SED as the spatial match was higher in the diaphysis on the periosteal surfaces. This was similar to the spatial match for models based on *ε*_maxprinc_ on the periosteal surfaces, but the spatial match was highest at the distal tibia on the endosteal surfaces. The spatial match in resorption was higher for models based on *ε*_maxprinc_ than SED on the endosteal surfaces, but the reverse was true for the periosteal surfaces. The variance was also larger for *ε*_maxprinc_ than SED.

The accuracy of remodelling was more consistent and higher for SED, but there was a higher variance due to a decrease in the predictive accuracy in the diaphysis (sections 4–6) for *ε*_maxprinc_. The spatial match of other tested parameters were very similar to the subject-specific results calibrated at week 20. The predictive accuracy was very low for resorption on both surfaces in the SED case, while *ε*_maxprinc_ captured resorption primarily in the diaphysis on both surfaces. The predictive accuracy for apposition and resorption mirrored each other for models based on *ε*_maxprinc_, but not for those based on SED.

## Discussion

In this paper, strain- and strain energy density-based bone remodelling algorithms for the murine tibia were developed and their outputs compared to longitudinal micro-CT images collected in vivo. The innovative parts of the study are represented by the definition and application of micro-FEA bone remodelling model in whole mouse tibia, the comprehensive comparison (validation) with longitudinal datasets, and the calibration of the mechanoregulation parameters for physiological loading.

The algorithm proposed in this study adopts a similar approach as other continuum models with tetrahedral meshes that used estimated strains at the bone surface to predict changes in shape (Cheong et al. 2018a; Pereira et al. 2015). Bone remodelling algorithms have been developed for both trabecular and cortical bone in micro-FEA, and applied to study osteoporosis and the effects of potential interventions in arresting trabecular thinning within the murine caudal vertebra (Schulte et al. 2011; Schulte et al. 2013b). In both models, the estimation of surface strains had an impact on the accuracy of the results and tetrahedral meshes have the advantage of capturing the surface geometry more accurately in modelling bone formation. However, voxel-based models of the mouse tibia have recently been validated against local measures with time-lapsed testing and digital volume correlation (Oliviero et al. 2018), demonstrating the feasibility to model cortical bone changes using micro-FEA.

The results showed that the parameters estimated from the optimisation algorithm were similar at weeks 14 and 20 of age (Table 2). This could be due to the small changes in SED between weeks 14 and 20 (Supplementary Fig. S2), the change in volumetric second moment between weeks 14–16 and 20–22 being in the same order of magnitude (Supplementary Fig. S3), and/or the similarities in densitometric measures between weeks 14–16 and 20–22 (Supplementary Fig. S4). There were some narrowing of the spread of densitometric parameters from weeks 16 to 22, which the bone remodelling algorithm was unable to account for (Fig. 3).

The apposition limits obtained in this study were approximately 3 times smaller than the peak *ε*_maxprinc_ calculated by the FEA model, and 10 times lower than the peak strain in tension measured using strain gauges on the diaphyseal mid-shaft of 12-week-old mouse tibia during walking (< 300 με) (de Souza et al. 2005). However, experimentally measured strains under mechanical loading of 11 N were approximately 1200–1500 με (de Souza et al. 2005; Willie et al. 2013). Scaling the model to an applied load of 11 N (as the FEA models used in this study were linear) would result in 1395 με, which falls within the range of values reported. The contribution of muscle load to the strain in the bone seems to be fairly large in the murine tibia and should be considered in future work.

Sub-analysis conducted on 40 compartments of the tibia showed that the choice of stimulus did not affect the shape of the errors in predicted and measured BV, BV/TV, BMC and BMD (Fig. 4). The over- (proximal end) and under- (diaphysis) prediction of the densitometric properties could be due to the differences in the amount of bone remodelling, as the proximal part and diaphysis are rich in trabecular and cortical tissues, respectively. Moreover, the differences between experimental measurements and computational predictions could also be due to the choice of metric (volumetric second moment) for the optimisation of the mechanoregulation parameters. The change in volumetric second moment was largest in the proximal regions, and this would have increased the weight for shape changes as the proximal tibia also has a higher number of voxels that are further from the neutral axis (Supplementary Fig. S3). The regional high errors for the predicted BV/TV in section 3 of the medial compartment were probably due to the pre-processing of images to virtually remove the fibula. Indeed, these cuts may not correspond exactly between the different time points. The highest absolute error was observed in section 10 (Fig. 4), where differences in apposition and resorption on both endosteal and periosteal surfaces were the largest (Fig. 5). The highest absolute errors in BV/TV for SED and *ε*_maxprinc_ using subject-specific parameters from weeks 20–22 were 10.3% and 8.1%, respectively. This compares favourably with other literature results which reported maximum prediction errors of 2.4% and 12.1% in BV/TV using SED, for murine caudal vertebrae under external mechanical loading, and ovariectomised (OVX) mice under physiological loading, respectively (Schulte et al. 2013b). The results obtained in this study for SED were also similar to their results in two other aspects, as there were no significant differences between the simulated and experimental densitometric indices, and the highest errors were located at the proximal end.

The highest standard deviations of the densitometric values (Fig. 4) were found in the trabecular region (section 10). This is consistent with literature findings as trabecular bone has a high turnover rate and a 2-week follow-up may be too long to obtain accurate point-to-point registration of trabecular bone changes (Webster et al. 2012). The use of average values instead of the subject-specific parameters calibrated from weeks 20–22 increased the densitometric errors in most cases, but there was no change to the location of the maximum predicted errors except for BMC in both SED- and *ε*_maxprinc_-based models, and BV/TV for *ε*_maxprinc_-based models. This could be due to the low volume of trabecular bone included in the model, as literature has reported that trabecular bone changes cannot be accurately predicted from averaged values from a population of bone scans (Webster et al. 2012). Moreover, the spatial match and predictive accuracy were not significantly different in the parametric study conducted, which suggests that future bone remodelling simulations in murine tibia involving only physiological loading can be calibrated from baseline scans and applied throughout the longitudinal study.

The experimental remodelling due to physiological loading (Fig. 5) showed that in week 14, resorption was more dominant on the periosteal surface than in week 16, but apposition on the endosteal surface was higher in week 14 than in week 20. This is similar to the results obtained from 26-week-old mice that underwent mechanical loading, which had higher bone remodelling on the periosteal surface at the proximal end, and on the endosteal surface at the diaphysis (Birkhold et al. 2017). In contrast, 3D fluorochrome mapping results obtained in a non-loaded contralateral control leg at week 22 showed that bone formation was located mainly on the endosteal surfaces (Carriero et al. 2018). The predicted overlap using subject-specific parameters from weeks 14–16 and 20–22 were similar, demonstrating the robustness of the model. The 59.1% and 47.4% spatial match in apposition and resorption using subject-specific, SED-based parameters values without a lazy zone from weeks 20–22 (Table 4) were similar to the results obtained by Schulte et al. (2013b) in the caudal vertebra. Their model used SED as the stimulus and achieved spatial match in 47.6% of the surface in apposition and 54.5% of the surface in resorption, for ovariectomised mice under physiological loading. Nevertheless, it should be noted that OVX models tend to show a decrease in trabecular BV/TV with age, contrary to the increase in trabecular BV/TV with age in healthy mice.

The 3D representation of bone remodelling predicted correctly on the periosteal surface showed that under physiological loading, bone apposition occurred mainly on the medial side, except at the proximal tibia crest (Fig. 6). On the lateral side, apposition occurred at the proximal and distal ends, while resorption occurred mainly between the tibial ridge and the interosseous crest in the diaphysis. Although the regions of remodelling generally matched with the SED distribution (Supplementary Fig. S2), it is slightly different from the bone remodelling observed in the murine tibia loading model, where bone formation has been reported to occur on the medial surface and the interosseous crest (Carriero et al. 2018; Pereira et al. 2015). Both SED and *ε*_maxprinc_ as the stimulus were able to predict bone apposition on both the endosteal and periosteal surfaces (Fig. 7), even though Carriero et al. (2018) reported that SED was able to capture external load-driven bone formation only on the periosteal surface. However, the spatial match for apposition was higher on the periosteal than the endosteal surface, for both mechanical stimuli (Fig. 8).

The prediction accuracy in resorption was very poor in SED. *ε*_maxprinc_ captured resorption and loss better than SED, but this was at the expense of apposition. The standard deviation of the prediction accuracy was also higher for *ε*_maxprinc_. These results could be due to the fact that *ε*_maxprinc_ is not an isotropic measure, and therefore more sensitive to orientation and registration. In addition, the algorithm uses a BRU lattice to compute the changes in bone density, which averages the signals of neighbouring voxels equally. Bone resorption has been reported to be more sporadically located across the length of the bone (Schulte et al. 2011), and the averaging approach may have masked the sites of isolated resorption. However, averaging is necessary in bone remodelling algorithm to maintain the continuum assumption in FEA models, and a weighted average could be considered as part of future work for regions where resorption are detected (Cheong et al. 2018a; Li et al. 2001). The strengths of SED and *ε*_maxprinc_ in predicting sites of apposition and resorption, respectively, also suggest that a combination of two or more stimuli may be required to improve the accuracy of the model, as the inclusion of tensile strain has been reported to increase the prediction of cortical bone changes (Carpenter and Carter 2008). Further sensitivity analysis on the choice of mechanical stimulus such as minimum principal strain should also be conducted.

This study has a number of limitations. Firstly, two small bending moments were induced and included in the analyses due to the geometry of the tibia under simulated compression. This was due in part to the application of the load through the centroid of the most distal slice, which may not correspond to the centre of motion at the ankle joint and thus affect the stimuli obtained. This distance is however very similar for each model thanks to the registration of the input micro-CT images to a reference bone. Nevertheless, further sensitivity analysis could be conducted to determine the effect the point of load application has on the small bending load induced. The prediction accuracy of the models (Fig. 7) was probably in part due to registration errors, as indicated by the large red patch of actual apposition that was not predicted, near the tibial edge at the distal end. Two sources of errors are the rigid registration of all bones to a reference bone, and the matching of each slice after centring the geometric centroid of the scans at different time points. The former introduces a rotational error, while the latter bias the alignment longitudinally by a fixed distance. Bone growth and formation are highest near the growth plates and decrease distally (Carriero et al. 2018), and an elastic registration should be considered as part of future work to map the locations of the bone with time. Moreover, the optimisation code uses the whole length of the tibia, in 10 approximately equal sections, for the calculation for the volumetric second moment. Sectioning may need to be region specific to capture changes in shape better. However, the current approach of registering the geometric centroid of the follow-up scans has the advantage of distributing the errors relatively equally across the 10 sections, compared with other 2D registration methods. Furthermore, the optimisation of the volumetric second moment may not give a unique solution, and although a grid of initial parameters was used to locate the global equilibrium, other parameters may be required to simulate resorption of bone primarily on the endosteal surface and the deposition of bone on the periosteal surface. Moreover, bone may only be partially optimised for loading conditions (Christen et al. 2012), and the physiological loading used in this study considered only the trotting motion, while other cage activities such as jumping and climbing have not been accounted for. Furthermore, a relatively simple structural homogeneous and isotropic micro-FEA model of the mouse tibia has been used. The study has only evaluated the performance of SED and *ε*_maxprinc_ as the stimulus. These stimuli would preferentially remodel regions that are under both compression and tension, or under tension, respectively. A more systematic approach by conducting detailed analyses of the strain (combined effects of different strain components) and strain gradient should be done to evaluate the choice of stimulus more comprehensively. The boundary conditions could be improved in future work, including the contribution of muscles and a spectra of possible physiological loading instead of a single loading scenario. Site-specific bone remodelling parameters could also be considered, as the change in volumetric second moment (Supplementary Fig. S3) and other literature have showed higher metaphyseal than diaphyseal bone remodelling (Birkhold et al. 2017). The apposition limits in the metaphysis and diaphysis are expected to be higher and lower than the values obtained in this study, respectively, but within the same order of magnitude. It should be considered that bone remodelling algorithms are primarily based on changes in mechanical stimulus, but in reality bone adaptation is due to a combination of mechanical and biological factors such as homeostasis, age and disease. Experimental studies have suggested that apposition is driven by mechanical factors, whereas resorption and loss may be driven more by biological factors (Birkhold et al. 2017; Schulte et al. 2013a). This would suggest either a stochastic approach for bone resorption, or a coupling with chemical or cellular models, as phenomenological models are limited in scale and scope. The use of fluid flow as a different mechanical mechanism has been reported to increase the predictive capabilities of the model on both endosteal and periosteal surfaces, and could be considered as part of future work (Carriero et al. 2018; Pereira et al. 2015; Villette and Phillips 2017).

In conclusion, in this study a novel algorithm for bone adaptation was developed to predict changes due to physiological loading. A phenomenological model was used with a lattice for bone remodelling unit to model changes in the grey values of the images, which was validated with in vivo longitudinal scans. The experimental and predicted results showed no significant changes in densitometric values, demonstrating the model’s capability to catch densitometric changes. The spatial match in apposition and resorption were similar for SED and *ε*_maxprinc_ as the stimulus. The predictive accuracy was above 50% in apposition, but very poor in resorption. Phenomenological models benefit from model simplicity and computational efficiency (Villette and Phillips 2017), but further work is required to improve the accuracy of the model in resorption, to make it applicable to study musculoskeletal diseases where prediction of resorption is important. The results showed that bone adaptation in murine tibia due to physiological loading can be estimated from a population of baseline scans and applied throughout the longitudinal study.

## Electronic supplementary material

Below is the link to the electronic supplementary material.

Supplementary material 1 (PDF 1253 kb)
