# Supplementary material for: A novel algorithm to predict bone changes in the mouse tibia properties under physiological conditions
Source: Biomech Model Mechanobiol. 2019 Nov 30;19(3):985–1001. doi: 10.1007/s10237-019-01266-7 (PMC7203598; doi:10.1007/s10237-019-01266-7)
Supplement: Supplementary file 1 — Supplementary material 1 (PDF 1253 kb) [file 10237_2019_1266_MOESM1_ESM.pdf]

A novel algorithm to predict bone changes in the mouse tibia properties under physiological conditions

Vee San Cheong [1,3], Ana Campos Marin [1,3], Damien Lacroix [1,3], Enrico Dall'Ara [2,3]

[1]Department of Mechanical Engineering, University of Sheffield, United Kingdom

[2]Department of Oncology and Metabolism, University of Sheffield, United Kingdom

[3] Insigneo Institute for *in silico* Medicine, University of Sheffield, United Kingdom

Corresponding author: V. S. Cheong (v.cheong@sheffield.ac.uk)

Supplementary Material

Table S1 Parametric analysis conducted on the inclusion of medial-lateral (ML) load in the bone remodelling algorithm

ML load	0		0.001BW	
Mouse	Remodelling rate, B (mg/cc-Pa-2weeks)	Apposition limit, k (Pa)	Remodelling rate, B (mg/cc-Pa-2weeks)	Apposition limit, k (Pa)
1	0.07	0.97	0.07	0.10
2	0.46	10.2	0.59	9.22
3	0.33	0.31	0.33	1.31
4	0.49	12.2	0.48	3.50
5	0.20	0.64	0.20	2.53
Average	0.31 ± 0.18	4.86 ± 5.83	0.33 ± 0.21	3.33 ± 3.53

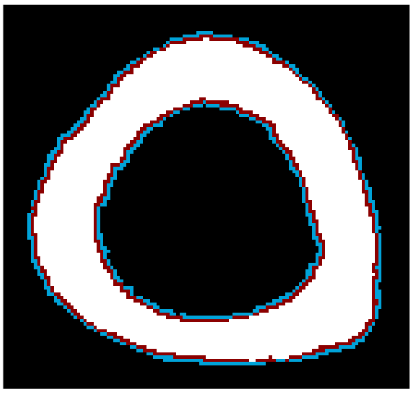

Fig. S1 An example illustrating the voxels in the background (blue) and bone (red) that were included in the transition zone (TZ).

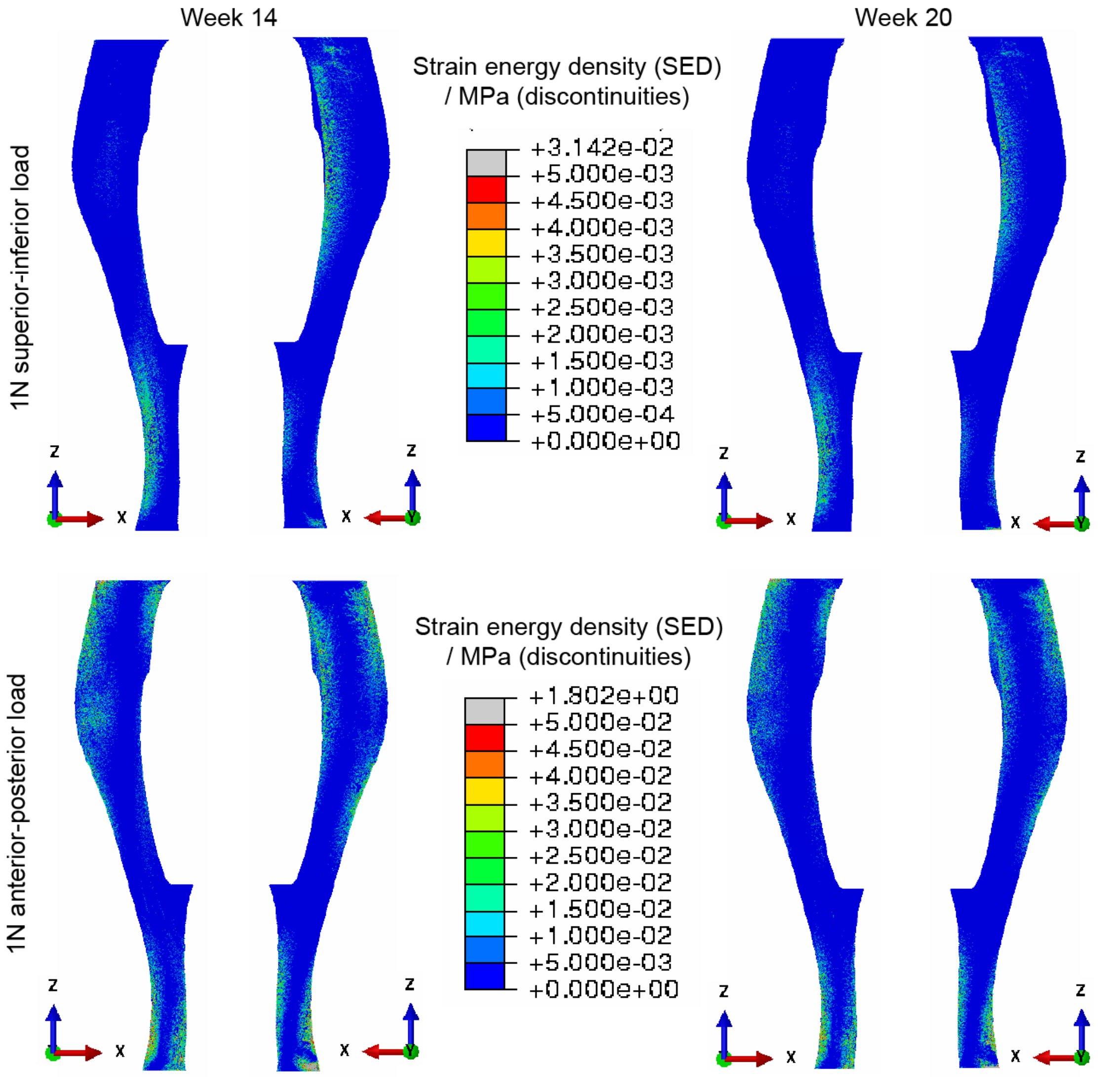

Fig. S2 Strain energy density distribution under 1N anterior-posterior load and 1N superior-inferior load for the right tibia of mouse 5 at weeks 14 and 20. The weight of 2 mice were higher at week 20 than 14, and for another 2 mice, was lower at week 20 than week 14. The weight of one mouse remained unchanged with age. Please note the difference in scale.

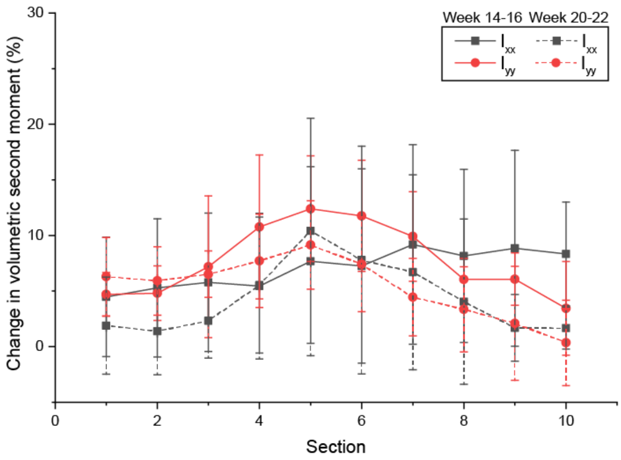

Fig. S3 Change in volumetric second moment between week 14-16 (solid line) and week 20-22 (dashed line) for I_{xx} (black) and I_{yy} (red).

A novel algorithm to predict bone changes in the mouse tibia properties under physiological conditions

Cheong et al.

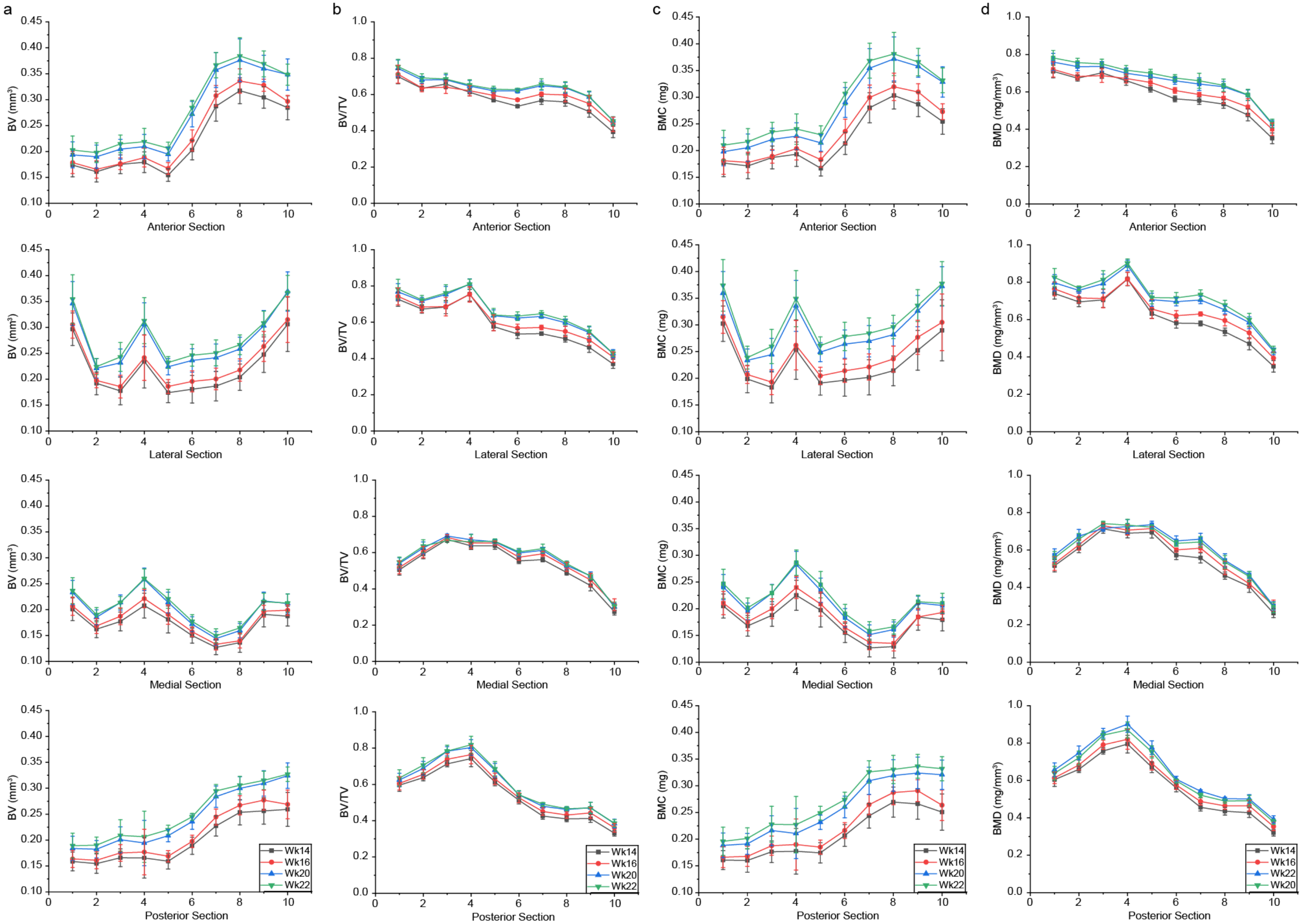

Fig. S4 Experimentally measured BV (a), BT/TV (b), BMC (c) and BMD (d) across the 40 partitions (four sectors from top to bottom; 10 sections) of the mouse tibia for weeks 14, 16, 20 and 22. X-axis indicates longitudinal sections from distal (0) to proximal (10).

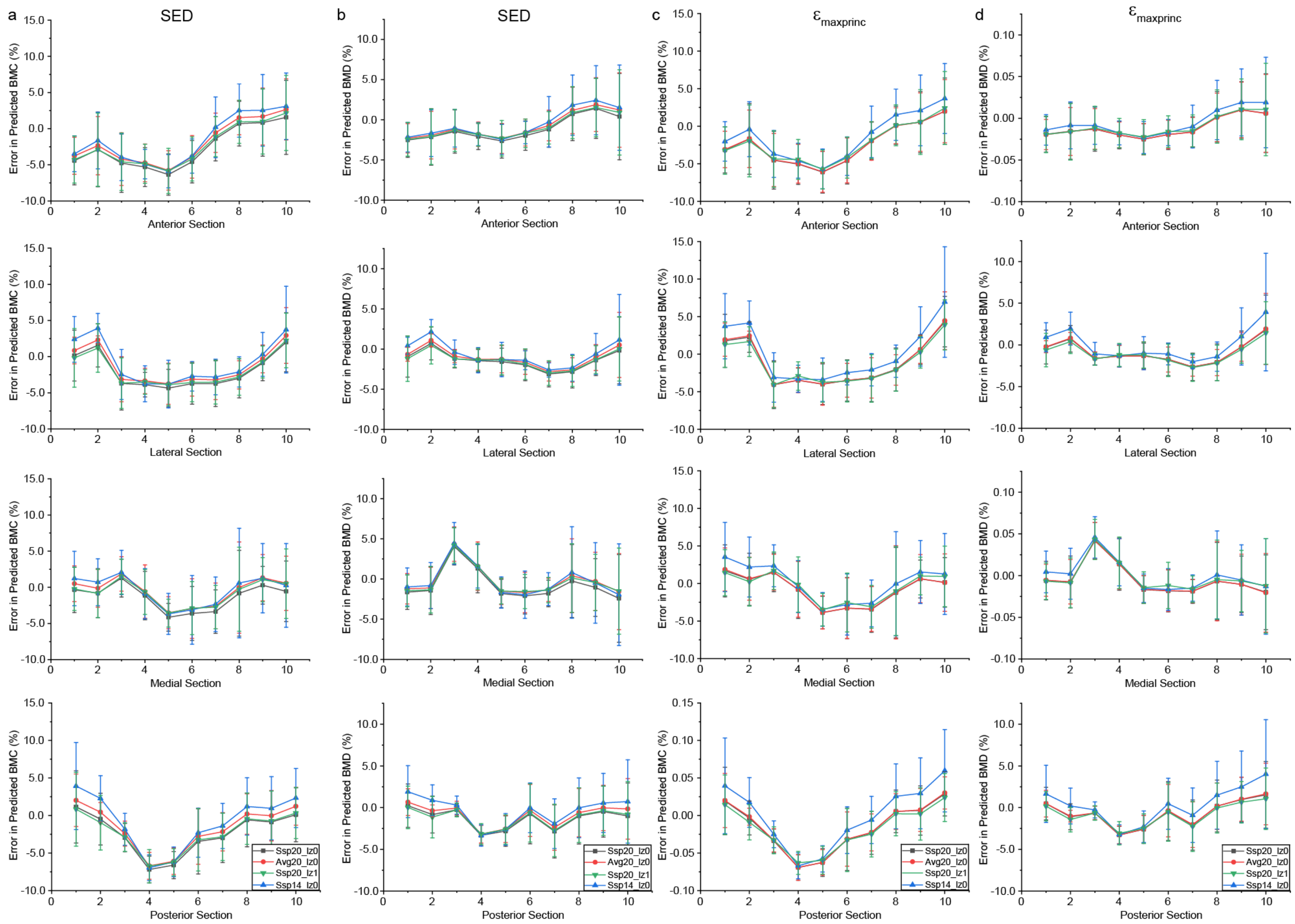

Fig. S5 Errors in predicted BMC and BMD for (a-b) *SED* and (c-d) $\varepsilon_{maxprinc}$ across the 40 sections of bone. SSp20_lz0: subject-specific parameters from weeks 20-22, no lazy zone. Avg20_lz0: averaged parameters from weeks 20-22, no lazy zone. Ssp20_lz1: subject specific parameters with lazy zone from weeks 20-22, no averaging. Ssp14_lz0: subject-specific parameters from weeks 14-16, no lazy zone. Numbers identify the mice in the experiments. X-axis indicates longitudinal sections from distal (0) to proximal (10).
